# Evaluation of preadipocyte factor-1 (Pref-1) level in cord blood of newborns born by mothers with gestational diabetes mellitus (GDM)

**DOI:** 10.1186/s12884-024-06517-9

**Published:** 2024-04-25

**Authors:** Monika Kolska, Justyna Agier, Elżbieta Kozłowska

**Affiliations:** Department of Microbiology, Genetics and Experimental Immunology, Centre of Molecular Studies on Civilisation Diseases, Medical University of Lodz, Mazowiecka 5 Street, Lodz, 92-215 Poland

**Keywords:** Pref-1 (DLK1), Gestational diabetes mellitus (GDM), Marker, Newborns, Cord blood, Metabolic syndrome

## Abstract

**Background:**

Gestational diabetes mellitus (GDM) is the most common metabolic complication, which leads to short and long-term consequences in both mother and fetus exposed to hyperglycemia. The aetiology of this condition is proposed to be based on the dysfunction of the adipose tissue, which is characterised by the aberrant generation of adipokines. One of them is preadipocyte factor-1 (Pref-1), which could mediate controlling the adaptation of the maternal metabolism to pregnancy.

**Aims:**

The study aims to examine the level of Pref-1 in the cord blood of healthy pregnant women’s neonates and fetuses born to mothers with GDM.

**Materials and methods:**

Cord blood samples were collected from 30 newborns of mothers with GDM and 40 newborns of healthy pregnant women. Pref-1 concentrations were measured with an ELISA kit.

**Results:**

Fetal Pref-1 concentrations were significantly lower in newborns of mothers with GDM compared to the normal pregnancy group children (5.32 ± 0.29 vs. 7.38 ± 0.53; *p* < 0.001). Mothers with GDM had a significantly higher index of BMI before pregnancy, maternal gestational weight gain, and maternal fasting glucose. In-depth analysis through multiple variant linear regression revealed a significant association between fetal serum Pref-1 levels, exposure to GDM, and gestational age.

**Conclusion:**

These findings contribute valuable insights into maternal-fetal health and pave the way for more targeted and effective clinical interventions.

## Highlights

Fetal Pref-1 concentration was significantly lower in GDM group.

Positive correlation between Pref-1 and exposure to GDM and gestational age

Pref-1 as one of mediators increased risk of metabolic diseases in late life.

Significant impact of intrauterine hyperglycemia on the offspring’s health.

## Introduction

Following the American Diabetes Association (ADA) [1], gestational diabetes mellitus (GDM) is an independent type of diabetes, which is defined as the first recognition of hyperglycaemia during pregnancy (usually between the 24th and 28th week of gestation). Of all pregnancies worldwide, GDM affects 1–36% of women and is the most common metabolic complication [2, 3]. The significant diversity in screening methods and diagnostic standards used to identify women with GDM makes it challenging to estimate and compare the incidence of this condition globally [4]. Most women diagnosed with GDM reside in the Asia-Pacific area. Asian inhabitants are more likely than Western cultures to be obese in the abdomen, have low muscle mass, and be more insulin resistant [5]. Most women with GDM have hyperglycemia resolved immediately postpartum, but they have a 17 to 63% risk of developing type 2 diabetes within 5 to 16 years after pregnancy. For this reason, the ADA recommends monitoring women with a history of GDM every three years, which could prevent or delay in the future the beginning of overt diabetes [6].

The aetiology of GDM relates to genetic and environmental factors. The first is usually called hereditary or demographic origins, non-modifiable risk factors. The second includes lifestyle behaviours during pregnancy, such as the maternal diet, including increased energy consumption and poor diet quality, increased glycaemic index foods in meals and low dietary fibre intake. Risk GDM has been implicated in pre-pregnancy obesity, gestational weight gain in early and mid-pregnancy, and physical activity habits during preconception and pregnancy. Furthermore, GMD is indicated to be associated with a shorter inter-pregnancy interval, smoking, and mental illnesses, including depression [7, 8]. A higher risk of developing GDM during pregnancy is associated with some endocrine disorders, such as polycystic ovarian syndrome (PCOS). A meta-analysis conducted by Qiu and colleagues [9] revealed that women with PCOS have an increased likelihood of developing GDM compared to women without this syndrome. This may be due to insulin resistance – a common feature of PCOS and GDM [10].

Effects of GDM are short and long-term complications in both mother and fetus exposed to hyperglycemia [11–13]. Pregnant women with GDM are at an increased risk of developing metabolic diseases, dyslipidemia, insulin resistance, and cardiovascular diseases, such as hypertension. The World Health Organisation (WHO) and the International Association of the Diabetes and Pregnancy Study Groups Criteria (IADPSGC) note neonatal complications in women with GDM, such as macrosomia, shoulder dystocia, respiratory distress, and higher perinatal mortality. Intrauterine hyperglycemia significantly impacts the offspring’s health. GDM causes an 8-fold increased risk of diabetes/ pre-diabetes (impaired glucose tolerance or impaired fasting glucose) in adulthood in the offspring of women with GDM [14, 15]. Studies have shown that children born to mothers with GDM have an increased risk of obesity and overweight in the future [16–18]. Infants born to mothers with GDM, particularly those with high birth weights, exhibit elevated lipid levels in the blood compared to the control group. Studies conducted in an animal model shed new light on the potential development of cardio-metabolic diseases in future generations of mothers with GDM. Considering the increased risk of diabetes and obesity in the offspring of women with GDM, it could be assumed that GDM in pregnant women may be one of the causes of intergenerational inheritance of cardiometabolic diseases [19–21]. Maternal diabetes during pregnancy has been associated with an increased likelihood of various psychiatric disorders in offspring, including schizophrenia, anxiety disorder, intellectual disabilities, developmental disorders, and behavioural disorders, as reported by findings from a Danish birth cohort study [22]. Research investigating cardiovascular and metabolic health among individuals with schizophrenia, including those who have not received treatment or taken antipsychotic medications before, has demonstrated significant evidence indicating a heightened risk for various cardiovascular conditions, diabetes, and metabolic syndrome [23, 24].

Adipose tissue dysfunction, characterised by abnormal adipokine production, might play a role in the pathophysiology of GDM [12]. Highly expressed in non-adipocyte cells in white adipose tissue is a transmembrane protein Pref-1 (preadipocyte factor-1). The protein is cleaved by a TNFα-converting enzyme to generate soluble forms, which act as an autocrine/paracrine factor [25, 26]. In the maternal circulation, a soluble, truncated form of the Pref-1, also named DLK1 (delta-like noncanonical notch ligand 1), could mediate controlling the adaptation of the maternal metabolism to pregnancy [27]. Pref-1 inhibits adipocyte differentiation and has been considered a molecular gatekeeper of adipogenesis [27, 28]. The origin of this protein may be both partially placental and fetal. Embryonic tissues, such as the lung, tongue, liver, hypophysis, developing vertebra, skeletal muscles, and adrenals, highly express Pref-1, reflecting its role in intrauterine development and growth. During the third trimester of pregnancy, the concentration of circulating Pref-1 rises, which reflects increased fetal weight [27].

Given the increasing rates of obesity and cardiometabolic disease, it is essential to focus on the pathogenesis of GDM to prevent them. Pref-1 could be one of the factors influential in the pathophysiology of GDM, so our research aimed to evaluate the concentration of this protein in cord blood newborns born by Polish women with GDM compared to newborns born by women without diabetes.

## Materials and methods

### Study groups

Thirty pregnant women with GDM and 40 pregnant women without any diabetes (control group) were recruited with the approval of the Bioethics Committee, Poland (RNN/596/14/KB) from the Polish Mother’s Memorial Hospital – Research Institute in Lodz. Patients with preexisting diabetes other than GDM, chronic generalised inflammation, immunosuppressive treatment, and/or end-stage malignant disease were excluded. All women have been informed about the aims and methods of the study and have expressed their written informed consent for participation in this study.

Before the pregnancy and in the 1st trimester, all participants consumed 400 µg of folic acid daily to prevent neural tube defects. Pregnancy was defined by a human chorionic gonadotropin (hCG) serum test level higher than 25 mlU/ml. An ultrasound was performed to confirm it and calculate gestational age.

In all patients, fasting blood samples were taken to measure plasma glucose, and then participants were subjected to an oral glucose tolerance test (OGTT) one hour and two hours post glucose load. GDM was diagnosed according to the criteria recommended by the IADPSG. GDM was defined as one or more elevated plasma glucose levels during a two-hour OGTT according to the requirements of the ADA. The following threshold plasma glucose levels were used: fasting: ≥5.1 mmol/l; one hour: ≥10.0 mmol/l; two hours: ≥8.5 mmol/l [14]. All GDM women controlled their glycemia with diet. The women in the control group had no gestational complications.

All participants underwent a thorough clinical examination, including anthropometric and laboratory measures. At the prenatal visit for 11–13^+ 6^ weeks, the pregnant women’s age, educational level, pre-pregnancy body weight, height, parity, and family history of diabetes were measured and written in the medical history. Body mass index (BMI) was determined as weight before gestation divided by squared height. Other parameters measured in women are maternal fasting glucose, mean glycosylated haemoglobin A1c (HbA_1c_) in 3rd trimester and smoking. Pref-1 concentrations, body weight, height, gestational age at delivery, fetal fasting glucose and Apgar points in 1st and 5th minute were measured in newborns.

### Laboratory measurements

Glycosylated haemoglobin A1c (HbA1c) levels were measured using high-performance liquid chromatography (HPLC). This method is certified by the National Glycohemoglobin Standardization Program (NGSP) and aligned with the International Federation of Clinical Chemistry (IFCC) reference method. The HbA1c test was used to diagnose diabetes, with a recommended threshold of ≥ 6.5% [29].

Umbilical blood samples were taken immediately after the delivery of the baby. Blood samples were centrifuged after standing at room temperature for at least 30 min. The serum was separated and stored at − 80 °C. Pref-1 concentrations were measured with a commercially available ELISA kit (Cloud-Clone Corp.). The minimum detectable dose of dLK1 was 0.257 ng/ml. The newborn blood glucose of all women was tested using the glucose oxidase method [14]. The first blood test was taken between 0 and 4 h of age, usually before the second feed.

### Statistical analysis

Arithmetical means and standard deviations (± SD), SEM, min. and max were calculated. The results were subjected to statistical analysis with STATISTICA software version 13. The Shapiro–Wilk test was used to determine the distribution. *U* Mann–Whitney or Student’s *t*-tests were used when two groups were compared, dependent on the type of distribution. To analyse the association of fatal Pref-1 with the presence of GDM, maternal age, gestational age at delivery, fetal gender and birth weight, multiple variant linear regression. The level of significance was determined (*p*). A *p* < 0.05 was statistically significant.

## Results

Table 1 presents the clinical parameters observed in women undergoing the study, comparing those with GDM and those with a normal pregnancy. While pregnant women with GDM tended to be older than those without GDM, however, the difference was not statistically significant (*p* = NS). These women also exhibited a significantly elevated BMI before pregnancy (*p* < 0.05), increased maternal gestational weight gain (*p* < 0.05), and higher levels of maternal fasting glucose (*p* < 0.05). Substantial differences were observed in maternal BMI before pregnancy, gestational weight gain, and maternal fasting glucose levels between the two groups – mothers with GDM and those with a healthy, normal pregnancy. Specifically, the differences were noteworthy, with the GDM group presenting with higher values for maternal BMI before pregnancy (26 ± 6.2 vs. 23 ± 4; *p* < 0.05), maternal gestational weight gain (11.1 ± 3.1 vs. 8 ± 1.4; *p* < 0.001), and maternal fasting glucose levels (86.5 ± 8.3 vs. 79.13 ± 7.7; *p* ≤ 0.001). These findings emphasize the distinct metabolic and anthropometric profiles in mothers with GDM, underscoring the importance of monitoring these parameters to understand the associated maternal health implications during pregnancy comprehensively.

**Table 1:** Clinical characteristics of mothers

	Mothers with GDM^a^ (n = 30)	Mothers with normal pregnancy (n = 40)	p
**Clinical data**			
Maternal age (years)	32 ± 4.8	30.5 ± 5	0.167
Maternal BMI (kg/m^2^) before pregnancy	26 ± 6.2	23 ± 4	0.039
Maternal gestational weight gain (kg)	11.1 ± 3.11 8 ± 1.4		0.000
Smoking, n (%)	2(6.7%)	4(10%)	-
Maternal fasting glucose (mg/dL)	86.5 ± 8.3	79.13 ± 7.7	0.001
Mean HbA_1c_^b^ (%NGSP) 3rd trimester Mean HbA_1c_ (mmol/molIFCC) 3rd trimester Mean HbA_1c_ (mg/dLeAG) 3rd trimester	5.04 ± 0.6 32 ± 4.2 98 ± 12.6	- - -	- - -

^a^GDM - gestational diabetes mellitus, ^b^HbA_1C_ - glycated haemoglobin is a form of haemoglobin that is formed in a nonenzymatic glycation pathway by haemoglobin exposure to plasma glucose

Table 2 provides an overview of the clinical parameters observed in newborns born to women with GDM compared to newborns of healthy women. Notably, no significant differences were identified in neonatal birth weight, length, Apgar scores at the 1st and 5th minute, paternal age, and gestational age at delivery between newborns of mothers with GDM and those with a normal pregnancy (*p* = NS). The average glucose levels in the newborns were within the range of 1.39–2.22 mmol/l. The second blood glucose test, administered between 4 and 24 h of age, revealed an average glucose level ranging from 1.94 to 2.5 mmol/l. Subsequently, the third blood glucose test, performed between 24 and 48 h of age, showed an intermediate glucose level of 2.5 mmol/l. These findings suggest that, despite maternal complications with GDM, the studied newborns did not exhibit significant differences in crucial clinical parameters when compared to those born to mothers with a normal pregnancy. The glucose levels within the specified time frames indicate a relatively stable metabolic profile in the newborns, reinforcing the need for ongoing monitoring and assessment in both groups.

**Table 2:** Clinical characteristics of newborns

	Newborns of mothers with GDM^a^ (*n* = 30)	Newborns of mothers with normal pregnancy (*n* = 40)	p
**Clinical data**			
Neonatal gender (n) female/male	13/17	18/22	-
Gestational age at delivery (weeks)	38.1 ± 1.8	37.7 ± 2	0.492
Neonatal weight (g)	3287 ± 476	3220 ± 489	0.708
Neonatal length (cm)	55.3 ± 2.6	54.7 ± 3.6	0.375
Neonatal fasting glucose (mg/dL)	67 ± 11	-	-
Apgar points in 1st minute	9 ± 1.4	9 ± 0,1	0.440
Apgar points in 5th minute	10 ± 0.9	10 ± 1	0.760
Age of fathers	35 ± 5	33 ± 5	0.327

^a^GDM- gestational diabetes mellitus

**Fig. 1:**
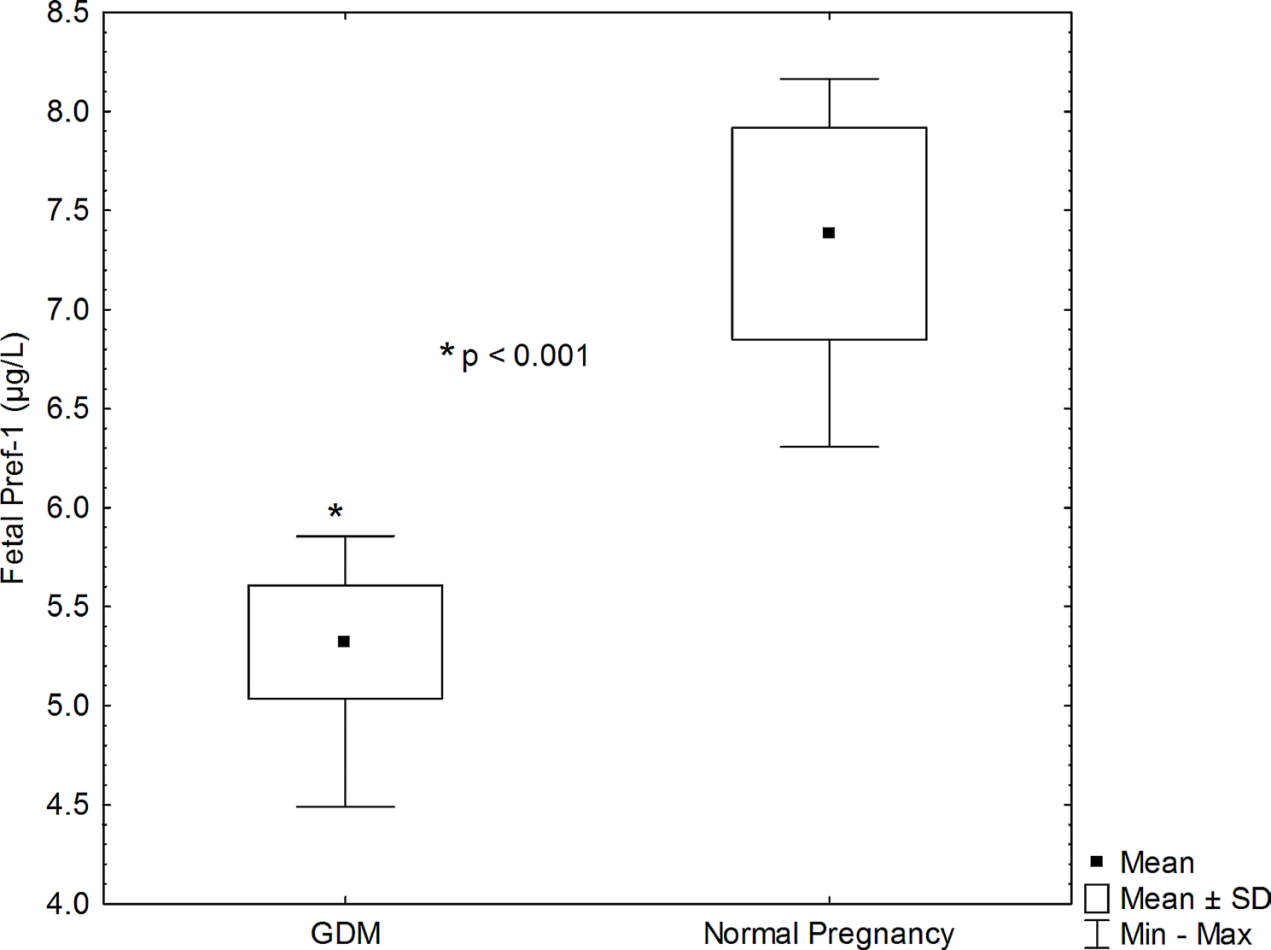
Comparison of fetal pref-1 between normal pregnancy and gestational diabetes. *There was a significant difference in fetal pref-1 concentration (p < 0.001)

The concentration of fetal Pref-1 was notably lower in the group of pregnancies complicated by GDM compared to those with normal pregnancies (5.32 ± 0.29 vs. 7.38 ± 0.53; *p* < 0.001), as illustrated in Tables 3 and in Fig. 1. In-depth analysis through multiple variant linear regression revealed a significant association between fetal serum Pref-1 levels, exposure to GDM, and gestational age (r2 = 0.473, *p* < 0.001). This association persisted even after adjusting for maternal age, fetal gender, and birth weight. Notably, when maternal age, fetal gender, and birth weight were considered in isolation, they did not significantly impact the observed association. These findings underscore the unique relationship between fetal serum Pref-1 concentration, exposure to GDM, and gestational age, highlighting the potential utility of Pref-1 as a biomarker in discerning the impact of GDM on fetal development during pregnancy.

**Table 3:** Neonatal pref-1 levels in study groups

	Study group Newborns of mothers with GDM	Control group Newborns of healthy mothers	p
**n**	30	40	
Pref-1 (µg/L) mean ±SD	5.32±0.29	7.38±0.53	*p* < 0.0001

GDM - gestational diabetes mellitus, ±SD - standard deviation

## Discussion

GDM is a hormone-triggered condition that disrupts the body’s capacity to use insulin effectively. Numerous adipokines, signalling proteins secreted by adipose tissue, have been identified as altered in individuals with GDM [30]. Understanding the role of adipokines in this condition is crucial for unravelling the complex interplay between pregnancy, hormonal changes, and glucose metabolism. Clarifying these connections could lead to more targeted interventions and preventive measures for pregnant individuals at risk of GDM.

In this study, we evaluated the concentration of Pref-1, which is closely related to insulin resistance, in the cord blood of mothers with GDM. Literature data on Pref-1 concentrations could be more extensive and explicit, especially in the context of GDM, highlighting the importance of further research. We detected in Polish women’s children that the Pref-1 level in cord blood was notably lower in the group of pregnancies complicated by GDM compared to those with normal pregnancies. These results are consistent with the observation from the Chinese group made by Li et al. [31]. They observed decreased serum concentrations of Pref-1 in GDM fetuses. Consistent with our findings, the authors demonstrated a relationship between gestational age and fetal Pref-1 concentrations. Furthermore, Çaltekin et al. [12] ascertained that pregnant woman diagnosed with GDM exhibited diminished levels of Pref-1 compared to their healthy counterparts within the Turkish population. A positive correlation was also observed between maternal serum Pref-1 levels and fasting insulin levels. Our investigation identified a positive association between maternal serum Pref-1 levels and fasting insulin concentrations.

Conversely, De Zegher et al. [32] reported a significant elevation in soluble Pref-1 levels among infants born small for gestational age (SGA) in the Belgian population, in contrast to control fetuses at birth. Notably, Wurst et al. [33] observed no discernible disparity in Pref-1 levels between pregnant women diagnosed with GDM and the control group within the German population. A noteworthy finding was the negative correlation between Pref-1 levels and BMI and C-reactive protein (CRP).

The decline in Pref-1 levels observed in our study aligns with the hypothesis advanced by Zhao et al. in the Chinese population [34]. Their investigation revealed that hyperglycemia in mothers diagnosed with GDM led to hypermethylation in the DNA region of the Pref-1 gene promoter. Consequently, there was a significant reduction in Pref-1 expression within the placenta of pregnant women with GDM. Furthermore, the methylation status of the Pref-1 gene on the maternal aspect of the placenta exhibited a robust correlation with maternal two-hour OGTT glucose levels. In contrast, the methylation status on the fetal side of the placenta demonstrated a pronounced association with fetal birth weight. This implies that perturbations in DNA methylation, induced by hyperglycemia, may disrupt regular Pref-1 expression in the placenta, potentially transmitting metabolic disorders such as obesity and insulin resistance across generations within the Chinese population [35]. Nonetheless, in the Danish population, evidence suggests that dysregulation in the expression of imprinted genes may contribute to postnatal metabolic disorders by influencing embryonic growth and development [36].

While Pref-1 is commonly recognised as an inhibitor of adipocyte differentiation within the American population [37], an alternative perspective emerges from a study indicating its potential to augment adipocyte differentiation in the English population [38]. Notably, Pref-1 expression is elevated in preadipocytes; nevertheless, as their differentiation progresses, there is a discernible decline in Pref-1 expression, ultimately culminating in its absence in mature adipocytes within the American population [39]. The susceptibility to GDM escalates with advancing age and the presence of obesity. According to Jensen et al. [40], there is an established correlation between Pref-1 and heightened obesity levels within the Danish population. This association may be attributed to the direct influence of Pref-1 on adipose tissue and its role in promoting insulin-dependent fat accumulation through the enhancement of insulin secretion in the pancreas. These American data are also corroborated by the study of Hudak et al. [41], which shows that Pref-1 ablation significantly reduces the development of white adipose tissue (WAT). Yet, these findings contradict studies, demonstrating that Pref-1-null mice are more obese in the USA [42] and that Pref-1-overexpressing mice are leaner [43]. This contradiction is attributed chiefly to the difference in body fat measurement techniques used in the studies [40]. However, this study did not find any correlation between BMI and Pref-1 concentrations.

Recent studies have highlighted the potential role of genetic factors in predisposing individuals to GDM [44]. Maternal lifestyle factors, such as diet and physical activity, may interact with genetic and hormonal factors to influence the development of GDM [45]. Obesity is an increasingly common condition among women, which exacerbates some genetic conditions such as PCOS. The pathophysiology of PCOS is diverse and involves many pathways and biomarkers. One is spexin (SPX), a hormone involved in different body organs’ metabolism and energy expenditure, which may serve as a connector between PCOS and GDM [46, 47]. Akbas et al. [47] revealed that serum spexin levels were significantly higher in women with GDM. It is worth analyzing the data presented by Mei et al. [26] that only a large soluble form of Pref-1 is biologically active. Moreover, alternative splicing determines Pref-1’s function in adipocyte differentiation, suggesting that comprehensive multicenter research efforts are needed to elucidate the multifaceted aetiology of GDM and the importance of Pref-1 in its development.

## Conclusions

In conclusion, the study delved into the concentration of fetal Pref-1, revealing a noteworthy protein level decrease in pregnancies complicated by GDM compared to normal pregnancies in the Polish population. The association between fetal serum Pref-1 levels, exposure to GDM, and gestational age persisted even after adjusting for confounding factors like maternal age, fetal gender, and birth weight. The pathophysiological significance of this regulation needs to be studied in more detail in future experiments. These findings may contribute valuable insights to the field of maternal-fetal health, paving the way for more targeted and effective clinical interventions.

The research has a few limitations. The first is a small research group of 30 pregnant women with GDM and 40 healthy pregnant women as a control group. A larger group size could provide more solidity and make generalisation possible. The second is that this investigation was conducted in the specific institute in Lodz. For this reason, these results can’t be translated to the broader population, especially considering potential differences in demographics or lifestyle. Moreover, the study did not consider the women’s BMI when collecting the material, which could shed new light on the obtained results.

## Data Availability

The datasets generated and analysed during the current study are not publicly available due to protect study participant privacy but are available from the corresponding author on reasonable request.

## Abbreviations

Pref: 1-preadipocyte factor-1
GDM: gestational diabetes mellitus
ADA: American Diabetes Association
PCOS: polycystic ovarian syndrome
IADPSGC: International Association of the Diabetes and Pregnancy Study Groups Criteria
DLK1: delta-like 1 homolog
hCG: human chorionic gonadotropin
OGTT: oral glucose tolerance test
BMI: body mass index
HbA1C: glycosylated haemoglobin A1C
HPLC: high-performance liquid chromatography
NGSP: National Glycohemoglobin Standardization Program
IFCC: International Federation of Clinical Chemistry
ELISA: Enzyme-Linked Immunosorbent Assay
IFCC: International Federation of Clinical Chemistry
HOMA_IR: Homeostasis Model Assessment of Insulin Resistance
CRP: C-reactive protein
SP: spexin
